# Risk Perception and PTSD Symptoms of Medical Staff Combating Against COVID-19: A PLS Structural Equation Model

**DOI:** 10.3389/fpsyt.2021.607612

**Published:** 2021-02-15

**Authors:** Qianlan Yin, Aibin Chen, Xiangrui Song, Guanghui Deng, Wei Dong

**Affiliations:** Department of Naval Aviation and Operational Psychology, Navy Medical University, Shanghai, China

**Keywords:** risk perceptions, PTSD symptoms, anxiety, sleep quality, PLS model

## Abstract

Medical staff were battling against coronavirus disease 2019 (COVID-19) at the expense of their physical and mental health, particularly at risk for posttraumatic stress disorder (PTSD). In this case, intervening PTSD of medical staff and preparing them for future outbreaks are important. Previous studies showed that perceived stress was related to the development of PTSD. Hence, in this study, the association between risk perception of medical staff and PTSD symptoms in COVID-19 and the potential links were explored. Three hundred four medical staff's exposure to COVID-19 patients, risk perception for working during COVID-19, PTSD symptoms, anxiety, and sleep quality were measured. Mediation analysis tested the indirect effects of anxiety and sleep quality on the relationship between risk perceptions and PTSD symptoms; 27.6% of participants were deemed as having probable PTSD diagnosis. Mediation analysis showed a significant chain-mediating effect of anxiety and sleep quality on the relationships between risk perceptions and PTSD symptoms; higher risk perceptions were related to increased anxiety, worsened sleep quality, and severe PTSD symptoms. Conclusively, medical staff have a high prevalence of PTSD symptoms after 3 months of COVID-19. Their PTSD symptoms were associated with the perceived risk level through the potential links with anxiety and sleep quality. Therefore, risk perception could be critical for our medical staff's responses to public health emergencies. It could be plausible to intervene in the perceived stress to alleviate aroused anxiety and improve sleep quality and thereby deter the development of PTSD.

## Introduction

The outbreak of coronavirus disease 2019 (COVID-19) has brought enormous physical and psychological pressure on Chinese medical staff. Medical staff often have various psychological problems under a high-pressure and risky antiepidemic situation (1). As reported, the overall incidence of medical staff's anxiety was 34.7% during the defense against COVID-19, which was obviously higher than before (2). Medical staff suffered more sleep problems than the general public (3). At the heart of the unparalleled crisis, medical staff must continue to successfully treat their patients and maintain personal responsibility, including taking care of their families and themselves. Therefore, the aggravated psychological burden leads to medical staff's burnout, insomnia, or even more serious problems (4).

### Post-Traumatic Stress Disorder Symptoms of Medical Staff

Medical staff are particularly at risk for posttraumatic stress disorder (PTSD) resulting from experiencing repeated or extreme exposure to aversive details of the traumatic event(s) (5). Against the background of COVID-19, medical staff are exposed to an increased danger of contamination, loss of patients, responsibility for difficult decisions on treatment retention, caring for severely traumatized people, frequent witnessing of death and trauma, interrupted circadian rhythms due to shift work, and disruption of normal supportive structures, all of which render medical staff more vulnerable to PTSD compared to the general population (6). A timely investigation showed the prevalence of PTSD symptoms 1 month after the outbreak of COVID-19 reached 3.8% in 377 Chinese medical staff (7). Meanwhile, a cross-sectional and survey-based study after the outbreak of COVID-19 on 1,778 healthcare workers (HCWs) and public servicers showed a total of 27.7% of the sample had clinical or subclinical symptoms of PTSD (8). The current COVID-19 pandemic is characterized by some relevant features that increase the risk for PTSD among medical staff involving the unprecedented numbers of critically ill patients, an unpredictable course of the disease, high mortality rates, and lack of effective treatment or treatment guidelines (9). Medical staff's suffering from a state of fatigue and rush for a long time could deteriorate their psychological and physical responses, some of which might revolve into the symptoms of PTSD.

### The Association Between PTSD and Risk Perception

As the literature documented, the unfamiliarity and perceived uncontrollability of the hazard involvement affect perceived risk levels and even affect a person's likelihood for developing PTSD (10–12). Moreover, the perception of life threat could predict PTSD after major trauma (13). In the epidemic of severe acute respiratory syndrome (SARS), researchers reported that levels of fear or having a sense of increase social isolation and (or) increased job stress related to SARS have previously been found to be varied with PTSD symptom levels (14–16). Respondents' perceptions of SARS-related risks were significantly positively associated with PTSD symptom levels and partially mediated the effects of traumatic exposure (17). Therefore, it postulated that risk perceptions of COVID-19 could influence PTSD symptom levels.

### The Definition and Measurement of Medical Staff's Risk Perception

Psychologically, risk perception refers to an individual's perception and understanding of various objective dangers in the outside world. The perceptions of risk are different in individuals facing the same situation. Also, people perceive various aspects of risk perceptions differently for emerging infectious diseases and tend to rate the situation as a risk, especially in the pandemic. Unfortunately, although risk perception has been well-studied in some areas, such as environmental risk, the medical staff's risk perception of COVID-19 has not been clearly defined and explored. A consensus is that the medical staff's risk perceptions refer to their knowledge, feelings, and understanding of risk factors and risk characteristics in the healthcare profession. A comprehensive study on risk perceptions of medical staff could be clustered into six dimensions: personal safety, physical function, occupational exposures, psychosocial concerns, organization safety, and timing pressure (18), which provides a dimensional model derived from risk perception for usual medical work. However, affected by the pandemic, medical staff felt their knowledge of the disease messed up, and their attitude and practice disrupted. As estimated, ~85% of the surveyed medical staff were afraid of becoming infected at work (19, 20). Although investigations have concerned the fear of being infected, a scarce study evaluated the dimensional risk perception of medical staff in epidemics. Therefore, the structured measure of risk perception could generalize the stress of medical staff.

### Anxiety and Sleep Quality Associated With the Risk Perception and PTSD Symptoms of Medical Staff

At present, no study explores these factors' effects on the relationship between risk perceptions and PTSD symptoms after the outbreak of COVID-19. Prior studies have confirmed that the exposure level, some social demographic factors, personality, and other individual characteristics are risk factors of PTSD (21, 22). However, little is known about which factors could affect the relationship between risk perceptions and PTSD symptoms. Emphasized by contemporary models in the etiology and maintenance of PTSD, anxiety is thought to be associated with a cognition of threat or danger (23). Similarly, a person with PTSD may come to have cognition of danger about stimuli (24). The risk perception can exacerbate or maintain certain PTSD symptoms (25). In a sense, a person feels increasingly anxious when misinterpreting signs of an impending threat and thereby exacerbates the PTSD symptoms. Additionally, the effects of anxiety on sleep quality have been investigated and proven by many studies (26–28). Meanwhile, shreds of evidence suggest that sleep is not simply a secondary symptom of PTSD but rather a risk factor for worsening symptoms of PTSD (29, 30). Therefore, it implies anxiety and sleep quality could be independent and interactive predictors of PTSD symptom severity. The outbreak of COVID-19 has turned studies to exploring stress, fear, anxiety, and symptoms of PTSD. These investigations showed that HCWs on the frontline more impacted by COVID-19 were at greater risk for anxiety and psychological disorder (31). However, a notable study measuring the vicarious traumatization phenomenon in three groups shows frontline nurses rated lower levels of trauma than the general public and non-frontline nurses (32), similar to findings discovered in 470 HCWs in Singapore, which showed non-medical workers had greater stress and anxiety than medical workers (6). These unexpected results highlighted the role of perceived risk and anxiety in the genesis of PTSD. Besides, Xiao et al. surveyed 180 medical staff and showed stress from the risk perception and anxiety were significantly associated, leading to negative impacts on sleep (33). However, the potential roles anxiety and sleep quality play in the relations between risk perceptions and PTSD symptoms during COVID-19 are still unknown.

### The Present Study

Learning about the risk perceptions, anxiety, sleep quality, and PTSD symptoms of medical staff during the epidemic may hold important lessons for handling future epidemics and understanding the psychological impacts of infectious disease outbreaks. Therefore, we carried out this explorative study on relations among these variables. Based on the previous studies, we hypothesized that against the backdrop of the COVID-19 epidemic, (1) higher level of risk perceptions was associated with severe PTSD symptoms; (2) risk perceptions were also associated with psychophysiological responses such as anxiety and sleep problems; and (3) these all directly or indirectly precipitate the symptoms of PTSD in the long term.

## Methods

### Participants

The current study was conducted on May 3–5, 2020, nearly 3 months after the peak of the epidemic, when the resuming progress was just ongoing in China. Medical staff were recruited from a general hospital with more than 500 employed medical staff in Shanghai. On the backdrop of an emergency, the temporary medical team joined in WeChat groups led by their administrators. We invited all the groups totaling ~400 medical workers to complete the online questionnaires through our provided website delivered in the chat groups. Eventually, 320 members submitted the questionnaires at the expiration date of our online investigation, and 304 responders (95%) were qualified to the criteria: (1) only the medical staff such as nurses, doctors, and medical technicians were recruited for the study, whereas the hospital administrators were excluded given that their job specifications are a bit different; (2) their time for completing the questionnaires were at a range of from 500 to 1,500 ms to avoid random fillings and unintentional or unintended answers; (3) no prior (to COVID) traumatic events, i.e., bereavement, divorce, or accidence. Among our subjects, 285 participants (93.7%) were nurses (4 were male nurses), 13 participants (4.3%) were male doctors, 3 (0.9%) were female doctors, and the rest, 3 (0.9%) participants, were male medical technicians. The average age of our sample was 30.42 ± 7.491 years, whereas that of 281 female nurses was 29.78 ± 7.074. All our participants were without traumatic exposures before the outbreak of COVID-19.

### Procedure

The research was evaluated and approved by the ethics committee of Navy Medical University in Shanghai, China. The written informed consents were obtained from the managers of the hospital, and consents of the medical staff were obtained from the e-mails. Data were collected using online versions of the questionnaires. All participants were informed that the researchers were interested in their experiences during COVID-19, and their participation was voluntary with anonymity. We contacted the hospital administrators and asked them to distribute the online questionnaires in their groups and emphasize anonymity and confidentiality. All the qualified questionnaires were scrutinized by our researchers according to selection criteria—being medical staff and finishing the whole questionnaires within the standard time range. Moreover, we consulted the prior traumatic history of these responders from the hospital's psychology departments.

### Measures

#### Demographic Questionnaire

This questionnaire included questions regarding gender, age, profession, the previous extent they contact the COVID-19 patients, and the current potential of being contacted.

#### Risk Perceptions Questionnaire

The questionnaire for risk perceptions was first formed through qualitative interviews and expert letter consultation by Zhang et al. (18). The initial items were based on a semistructured interview with the nursing staff. Combined with the results of two rounds of consulting experts, the items were strictly modified, deleted, and merged to ensure good content validity. Finally, the reliability and validity of the questionnaire were evaluated by multiple analyses. In deference to our research design, this questionnaire was adapted for a short version. The self-rated questionnaire includes 14 questions pertaining to six dimensions: personal safety risks: threats to life or body usually caused by irrational patients (questions 1–2), physical function risks: injuries to physical health from illnesses (questions 3–4), occupational exposure risks: exposures to virus and injuries from operations (questions 5–7), psychosocial evaluation risks: stigmas and other social judgments (questions 8–10), organizational risks: threats to self-interest caused by hospital regulations and managements (questions 11–12), and time pressure: limited time for own pleasures (questions 13–14). The rating potentiality of risk was measured by self-reported frequencies of being worried or threatened by the risk, which is a superior indicator of risk perception rather than probability (34). The frequencies were divided into five grades from “never” to “always” and assigned 1 to 5 points relatively. The higher the score for each dimension, the higher the level of medical staff's specific risk perception. However, one point that should be emphasized here is that the concept of risk perceptions is formative, different from traditionally reflective items, which could be coexisted and converged into one concept (35–37). As a formative measurement model, each dimension of risk perceptions is not necessarily correlated and coherent. For example, if the personal safety risk is perceived to be at a high level, the susceptibility to physical function risk could be high or low. Therefore, it is not necessary for reporting the total consistency coefficient of a formative measurement model, but the dimensional scales. In our study, Cronbach α coefficient of each adapted subscale ranged from 0.768 to 0.854. An English version of the adapted questionnaire was attached as Supplementary Materials.

#### The PTSD Checklist for DSM-5

The PTSD Checklist for *Diagnostic and Statistical Manual of Mental Disorders, Fifth Edition* (*DSM-5*) (PCL-5) was developed by an American PTSD research center following the *DSM-5*. This scale includes 20 items for evaluating four clusters of PTSS, including intrusive symptoms (criterion B: questions 1–5), avoidance symptoms (criterion C: questions 6 and 7), negative alteration in cognition and mood (criterion D: questions 8–14), and hyperarousal symptoms (criterion E: questions 15–20). Respondents were asked to rate how bothered they have been by each of 20 items in the past month on a 5-point Likert scale ranging from 0 to 4. Items are summed to provide a total severity score (range = 0–80). The total PCL, criterion B, criterion C, criterion D, and criterion E had Cronbach α coefficients of 0.906, 0.798, 0.791, 0.750, and 0.829, respectively, in this study, thereby indicating high internal consistency. The PCL-5 can determine that a provisional prediction according to summing all 20 items and using the cutoff point score of 33 for PTSS appears to be reasonable based on current psychometric work, considering the goals of the assessment and the population being assessed and giving a probable differentiation (38).

#### Generalized Anxiety Disorder Scale

A Chinese version of Generalized Anxiety Disorder-7 (GAD-7) scale was used to assess the subject's anxiety symptoms. The GAD-7 has been previously used in Chinese populations and was found to have good reliability (Cronbach α = 0.90) (39). Seven items assess the frequency of anxiety symptoms over the past 2 weeks on a 4-point Likert scale ranging from 0 (never) to 3 (nearly every day). The total score of GAD-7 ranged from 0 to 21, with increasing scores indicating more severity resulting from anxiety (40). In our study, Cronbach α was 0.918.

#### Pittsburgh Sleep Quality Index

The Pittsburgh Sleep Quality Index (PSQI) is a self-administrated questionnaire that assesses sleep quality and disturbances over a 1-month time interval (41). The Chinese vision of PSQI was also proven to be valid by Liu and Tang (42). It is indicated that the use of “component” scores in the PSQI is also empirical and clinical (43, 44). Therefore, we selected five questions from PSQI in the present study, including subjective sleep quality, asking “How good is your sleep quality?”; sleep disturbances, asking “Do you have easy waking during sleep and early waking in the morning?”; sleep latency, asking “Do you have difficulty in starting sleep?”; sleep duration, asking “What's your actual sleep time recently?”; and energy, asking “Have you had a lack of energy for the past month?” Each question weighted equally on a 0- to 3-point scale. The scores of five questions were summed to yield a global PSQI score; higher scores indicate worse sleep quality. Cronbach α coefficient of the global score was 0.821 for this study.

### Statistical Analysis

Data screening showed there were no missing values for the questionnaires. SPSS 24.0 statistical software was used for data processing and analysis for description and correlation. The mean and standard deviation (SD) of the statistical score data were reported. Non-parametric test and χ^2^ test were adopted for exploring the difference between groups with or without probable PTSD. The partial least squares (PLS) (45, 46) method was employed to estimate the mediating model. PLS is a second-generation structural equation modeling (SEM) technique developed by Wold (47). It works well with structural equation models that contain latent variables and a series of cause-and-effect relationships (48). PLS has three major advantages over other SEM techniques that make it well-suited to this project. First, in PLS, constructs may be measured by a single item, whereas at least four questions per construct are required in covariance-based approaches. Second, the results from a PLS analysis can also be arguably said to be easier to assess. Rather than determining whether various model fit indices are appropriate, PLS focuses on variance explained (i.e., the predictiveness of the model) (45). Importantly, PLS can model formative measurement items, while using SEM analysis can lead to identification problems (49). PLS employs bootstrapping to test the significance of relationships; therefore, PLS may perform well in testing mediation effects (50). It outlined the product of coefficients involving paths in a path model approach. The path coefficients generated by PLS indicate relationships and can be used similarly to the traditional regression coefficients (51). Paths that are non-significant or show signs contrary to the hypothesized direction do not support a prior hypothesis, whereas significant paths showing the hypothesized direction empirically support the proposed causal relationship. In this study, bootstrapping with 2,000 retrials was used to evaluate the significance of the path coefficients and estimate the standard error. Meanwhile, the primary evaluation criteria for the structural model are the *R*^2^ measures because the goal of the prediction-oriented PLS-SEM approach is to explain the endogenous latent variables' variance; therefore, the key target constructs' level of *R*^2^ should be high. On the specific research discipline, *R*^2^ values of 0.75, 0.50, or 0.25 for endogenous latent variables in the structural model can be described as substantial, moderate, or weak, respectively.

## Results

### Descriptive Results

Descriptions for traumatic experiences, dimensional risk perceptions, PTSD symptoms, anxiety, and sleep quality are presented in Table 1. As for traumatic experiences, 78% of our sample recalled that they did not contact confirmed COVID-19 patients, whereas 17% had contacts with those patients at the peak of the epidemic. However, 3 months after the peak time, 55% of our sample were thought they are likely to be contacted, and 9% believed they have many possibilities of infection, whereas 36% of our cohort believed that they were not likely to contact the patients. Furthermore, 114 participants reported their current contacts with the infected patients were more than before; conversely, 96 people reported the opposite. Generally, resuming their work after the peak of COVID-19, medical staff continued fearing for the virus and perceived the situation as a risk. As ratings for dimensional risks showed, only the organizational risk was averaged <2.5 points); the other five risks were recognized as frequent by our sample, especially the time pressure. In terms of PTSD symptoms, the C cluster for avoidant symptoms was rated lower; E for hyperarousal symptoms was rated relatively higher, suggesting the hyperarousal characteristic could be easily seen in our sample.

**Table 1:** Means and standard errors for subscale and total scores of questionnaires.

**Questionnaires**	**Variables**	**Mean**	**SD**
Traumatic experiences(4-point scale)	Previous contact	1.59	1.15
	Potential contact	1.74	0.661
Risk perceptions(5-point scale)	Personal safety risk	2.65	0.967
	Physical function risk	2.94	0.932
	Occupational exposure risk	2.49	0.833
	Psychosocial evaluation risk	2.67	0.902
	Organizational risk	2.41	0.847
	Time pressure	3.03	1.15
PCL-5(5-point scale)	Criterion B	0.441	0.977
	Criterion C	0.151	0.491
	Criterion D	0.490	1.15
	Criterion E	0.641	1.17
	Total score	28.9	9.18
GAD-7 (4-point scale)	Anxiety	9.88	3.39
PSQI (4-point scale)	Sleep quality	7.79	2.68

### Differences Between Medical Staff With or Without Probable PTSD

Eighty-four of 304 participants could be identified with probable PTSD using the cutoff point of PCL-5; thus, the prevalence of probable PTSD was 27.6% in our sample. Furthermore, participants were grouped into a without-PTSD group and a with-probable-PTSD group. As shown in Table 2, there were significant differences between the two groups in risk perceptions, anxiety, and sleep quality, but no difference in aspects of exposure experiences and age. However, the actual possibility of medical staff's infection seemed to be unrelated to PTSD symptoms as a χ^2^ test showed that previous and potential contacts with the COVID-19 patients were disparate in the two groups (χ^2^ = 3.467, *p* = 0.084 > 0.05). Next, a correlational analysis was conducted on variables different in the groups, showing that all specific risk perceptions were positively related to anxiety, sleep quality, and PTSD. Meanwhile, each pair correlation among anxiety, sleep quality, and PCL-5 scores was significant. All the coefficients of associations are presented in Table 3.

**Table 2:** Differences in measured variables between medical staff with or without probable PTSD.

	**Probable PTSD (*n* = 84)**	**Without PTSD(*n* = 220)**	***Z***	***p***
Criterion B	1.17 ± 1.50	0.163 ± 0.428	7.07	0.000
Criterion C	0.476 ± 0.799	0.027 ± 0.189	7.04	0.000
Criterion D	1.43 ± 1.80	0.132 ± 0.365	8.89	0.000
Criterion E	1.61 ± 1.72	0.273 ± 0.522	7.41	0.000
Total score	41.5 ± 7.06	24.1 ± 3.67	13.5	0.000
Age	29.7 ± 7.16	30.7 ± 7.61	0.866	0.386
Previous contact	1.40 ± 0.983	1.66 ± 1.21	1.48	0.138
Potential contact	1.82 ± 0.679	1.71 ± 0.653	1.32	0.189
Personal safety risk	3.16 ± 0.858	2.46 ± 0.938	6.00	0.000
Physical function risk	3.39 ± 0.889	2.77 ± 0.892	5.01	0.000
Occupational exposure risk	2.86 ± 0.836	2.35 ± 0.788	4.81	0.000
Psychosocial evaluation risk	3.13 ± 0.859	2.50 ± 0.858	5.49	0.000
Organizational risk	2.79 ± 0.945	2.27 ± 0.761	4.74	0.000
Time pressure	3.57 ± 1.09	2.82 ± 1.10	5.04	0.000
Anxiety	12.7 ± 3.62	8.81 ± 2.60	6.52	0.000
Sleep quality	9.48 ± 2.80	7.15 ± 2.34	9.07	0.000

**Table 3:** Correlations between risk perception, anxiety, sleep quality, and PTSD symptoms.

	**1**	**2**	**3**	**4**	**5**	**6**	**7**	**8**	**9**
1. Personal safety risk	1								
2. Physical function risk	0.770^**^	1							
3. Occupational exposure risk	0.650^**^	0.724^**^	1						
4. Psychosocial evaluation risk	0.640^**^	0.628^**^	0.697^**^	1					
5. Organizational risk	0.512^**^	0.559^**^	0.631^**^	0.622^**^	1				
6. Time pressure	0.391^**^	0.480^**^	0.429^**^	0.527^**^	0.536^**^	1			
7. Anxiety	0.438^**^	0.475^**^	0.452^**^	0.431^**^	0.441^**^	0.410^**^	1		
8. Sleep quality	0.301^**^	0.362^**^	0.285^**^	0.279^**^	0.238^**^	0.276^**^	0.347^**^	1	
9. PCL-5 scores	0.365^**^	0.376^**^	0.326^**^	0.371^**^	0.300^**^	0.325^**^	0.575^**^	0.475^**^	1

** *p < 0.01*.

### Explorative Analysis of the Correlated Variables and Their Intertwined Relationships

**Figure 1:**
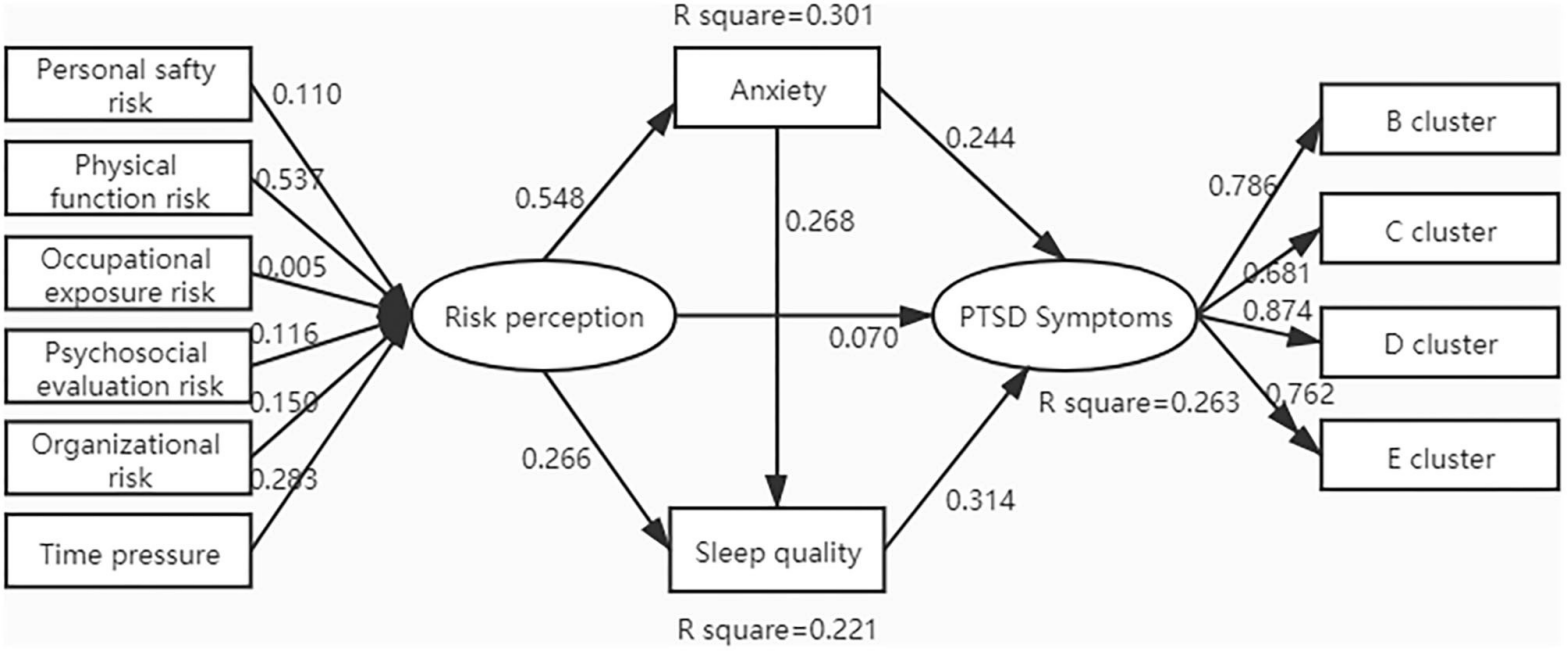
The chain-mediating effect of anxiety and sleep quality on the relationship between risk perceptions and PTSD symptoms using a PLS model.

Based on the cognitive and behavioral theory and previous studies mentioned in the introduction, medical staff's risk perception affected by the epidemic outbreak could leverage their anxious emotion and worse sleep quality and then evolved the PTSD symptoms as a behavioral outcome. To prove the hypothesis, the chain-mediating effect using a PLS model was proposed and tested. Only the female nurses'data have entered the model (including 281 participants with an average age of 29.81 ± 7.112) for controlling the effects of other factors such as gender and profession. The final model is shown in Figure 1, which illustrates that the total adjusted *R*^2^ for PTSD symptoms is 0.263. The collinearity statistics for variance inflation factor (VIF) of all variables ranged from 1.312 to 3.407, meeting the criterion of <5. This model shows anxiety and sleep quality play a chain-mediating role between risk perceptions and PTSD symptoms. Illustrated in Table 4, the total effect of risk perceptions on PTSD symptoms was significant, and the coefficient was 0.333 (*p* < 0.000), but the direct effect of risk perceptions on PTSD symptoms was not significant (*p* = 0.304). However, the specific indirect effects were all significant as all the 95% confidence intervals of the bootstrap test did not include zero, also the chain-mediating effect through anxiety and sleep quality (β = 0.046, 95% confidence interval = 0.020–0.077, *p* < 0.000), statistically indicating anxiety and sleep quality could completely mediate the effect of risk perceptions on PTSD symptoms. Moreover, the weight of personal function risk on risk perceptions was 0.537, and compared to other risks, it tended to be the major contributor to the emotion of anxiety and sleep quality. Accordingly, the prediction of PTSD symptoms concerted on the D cluster (β = 0.874), which includes more symptoms of negative alteration in cognition and mood. Notably, considering establishing this ordering of mediation model, we tested alternative models: first, alternative model 1 run the model in the opposite direction of the model presented in the figure, attaining that the total adjusted *R*^2^ for risk perception was 0.348, but contributions to anxiety and sleep quality were weakened (the adjusted *R*^2^ values were 0.170 and 0.261 relatively); second, alternative model 2 changed the direction of anxiety and sleep quality, showing a slight decrease in the regression weight of path between anxiety and sleep quality, and path through risk perception to PTSD symptoms. As proven, the final model presented in the figure was better fitted in aspects of path coefficients and levels of *R*^2^, which also confirmed the previous hypothesis. Meanwhile, we explored the model with the total sample and the other model with controlling the variable of age; results showed changes were detected in the regression weights but were not in the significant coefficients of paths (see the Supplementary Materials for the detailed testing results for all models).

**Table 4:** Path coefficients and the confidence intervals.

	**β**	**SD**	**Lower bounds**	**Upper bounds**	***t***	***p***
**Total effect**						
Anxiety → PTSD symptoms	0.329	0.083	0.153	0.478	3.972	0.000
Anxiety → sleep quality	0.268	0.066	0.136	0.391	4.094	0.000
Risk perceptions → anxiety	0.548	0.049	0.453	0.645	11.162	0.000
Risk perceptions → PTSD symptoms	0.333	0.055	0.243	0.455	6.111	0.000
Risk perceptions → sleep quality	0.413	0.046	0.332	0.517	8.930	0.000
Sleep quality → PTSD symptoms	0.314	0.068	0.172	0.438	4.596	0.000
**Direct effect**						
Anxiety → PTSD symptoms	0.244	0.083	0.077	0.395	2.942	0.003
Anxiety → sleep quality	0.268	0.066	0.136	0.391	4.094	0.000
Risk perceptions → anxiety_	0.548	0.049	0.453	0.645	11.162	0.000
Risk perceptions → PTSD symptoms	0.070	0.068	−0.047	0.219	1.028	0.304
Risk perceptions → sleep quality	0.266	0.066	0.148	0.403	4.041	0.000
Sleep quality → PTSD symptoms	0.314	0.068	0.172	0.438	4.596	0.000
**Specific indirect effects**						
Risk perceptions → anxiety → PTSD symptoms	0.134	0.051	0.039	0.235	2.634	0.009
Risk perceptions → anxiety → sleep quality → PTSD symptoms	0.046	0.015	0.020	0.077	3.149	0.002
Risk perceptions → sleep quality → PTSD symptoms	0.083	0.030	0.035	0.149	2.808	0.005
Risk perceptions → anxiety → sleep quality	0.147	0.040	0.072	0.228	3.714	0.000

## Discussion

The overall purpose of this study was to develop an understanding of the relationship between risk perceptions, anxiety, sleep quality, and PTSD symptoms of medical staff on the background of COVID-19. The prevalence of probable PTSD in our sample was estimated to be 27.6%. PTSD symptoms were positively related to individual risk perceptions. A major concerning risk was time pressure; the commonest PTSD symptom was hyperarousal according to the average score for self-ratings, whereas in the predictive analysis, their physical function risk perceptions contributed most to risk perceptions and may well predict the cognitive symptoms of PTSD. Anxiety and sleep quality were coexistent and interrelated in mediating the relationships between risk perceptions and PTSD symptoms.

Studies of risk perceptions and PTSD in epidemic settings are new. To our knowledge, this is the first study to examine the prospective relationships between risk perceptions of medical staff and PTSD symptoms after 3 months of COVID-19. In our cohort, the prevalence of PTSD was 27.6%, equal to the result from a mental health survey of 230 medical staff in a tertiary infectious disease hospital (52). Compared to the studies in the early period of COVID-19 (6, 7), the prevalence was relatively higher because of the development of PTSD symptoms and the persistent risks. As reported, symptoms persisting for more than 6 months after an event are likely to persist over the long term (53, 54). In this case, we should take immediate intervention for PTSD resulting from COVID-19 and deter its development. Notably, during the COVID-19 outbreak, the PTSD symptoms in our cohort were not discriminated in the traumatic exposures defined by the extent of being exposed to COVID-19; instead, the perceived risks were differentiated across the severity of PTSD symptoms. Inconsistent with previous studies, we did not find contact frequency as a risk factor for PTSD (7, 55). It partly lied in that our sample was collected by cluster sampling from the same hospital in Shanghai, which was not the hardest-hit area and well-defensed for COVID-19. However, in the resuming periods of China, Shanghai continued having imported cases and being tightly supervised. In this case, media reports centered on transmitting the information focused on imported cases, which turn an international city like Shanghai to be the focus. This also partially explained why our sample was alert to risk and perceived the frequent potential contacts. Moreover, with the traumatic event less varying and exposure level controlled, the effect of risk perceptions on PTSD symptoms could be brought out.

Results show high levels of perceptions for each dimensional risk, especially the physical function risk, could increase the negative emotions of anxiety, worsen sleep quality, form the negative metacognition, and sequentially develop the medical staff's PTSD symptoms (56, 57). Physical functions of medical staff suffered from overwhelmed works and insufficient protection caused by COVID-19. Compared to other risk perceptions during the COVID-19, physical function risk strongly contributed to the symptoms of PTSD such as hyperalertness and emotional avoidance. It could be a normal stress reaction for nurses to worry about their physical health threatened by the transmittable virus. However, when the worries persisted or worsened to be anxiety or fears, in a severe sense, they could affect sleep quality and presented as sleep disturbance, early wakes, or nightmares. If these worries were not relieved or properly copied, consequentially, they could generate PTSD symptoms. Consistent in previous clinic studies (58, 59), the effects of anxiety and sleep quality on PTSD of nurses suggest that anxiety and nightmares are specific work-related triggers for symptoms of PTSD. Anxiety was reported to be related to perceptions of being overextended at work in more than 50% of nurses (60). The novel chain–mediating model also presented the relations between risk perceptions and psychophysical reactions such as anxiety and sleep quality, based on the hypothesis from the perspective of cognition–emotion–behavior theory (61). However, it could be possible that anxiety measured as the general anxiety might persist and overlap with the arousal symptom cluster of PTSD, which added to a significant chain effect on hyperarousal symptoms presented in the final model. Therefore, indirect relations between risk perception and PTSD via anxiety and sleep quality need to be further studied.

Our findings complement studies of nurses' burnout and PTSD symptoms, which proposes that PTSD and burnout have commonly coexisted in nurses (60). In the work environment, nurses with PTSD and burnout have more negative opinions regarding the people they work with, thereby increasing the risk perception of the organization management and affecting their psychosocial support systems (62). Notably, young female nurses, who are the core of their family and have to balance their time for work and family, under the impact of the virus, need to devote more time to the work, take more protective but fussy measures, and keep a distance from their relatives and lovers. Collectively, they are burned out physically and mentally in signals of their risk perceptions for time pressure and lacking psychosocial supports.

COVID-19 had brought a whole new set of challenges to medical staff with great psychological impacts. As the pandemic continues, important clinical and policy strategies are needed to support them. Our study identified medical staff is a vulnerable group susceptible to PTSD symptoms. Risk perceptions affect their symptoms by increasing anxiety and worsening sleep quality. Hence, education inventions should target medical staff to ensure understanding and use of infectious control measures, which could help staff reasonably perceive the potential risks. Meanwhile, their personal safety should be ensured and strengthened by providing reliable protective controls and isolation at the same time with psychological support like counseling services. Emphatically, psychological help and cognitive–behavioral therapies for relieving anxiety are imperative and effective for intervening in PTSD symptoms of medical staff.

The present study builds the chain-mediating model by PLS analysis, which allows studying the formative measure structure of risk perception and its associations with PTSD. Weight indications and the significant direct and indirect effects proved the cognition–emotion–behavior links for PTSD development, suggesting that cognitive–behavioral treatments for PTSD should be more tailored to medical staff targeted at risk perception. There are several limitations to our findings. First, the cross-sectional nature limited the establishment of a causal relationship between risk perception and PTSD symptoms. Although the PLS analysis can be stronger in predicting dependent variables than SEM, longitudinal designs and studies are still needed to confirm the relation, especially the chain effect of anxiety and sleep quality, given that a retrospective reporting bias may contaminate this attempt to establish temporal precedence between constructs. Second, the small sample size and the local sampling should be notified. On the one hand, a homogenous sample of nurses within a single hospital setting in an international city facilitated control of other related factors such as gender and occupation. On the other hand, it would decrease the reliability and generalized findings. Besides, the possible self-selection bias associated with the online method should be acknowledged as a limitation. Moreover, one should be cautious about interpreting the completely mediating role of anxiety and sleep quality as more other latent variables (i.e., depression, fear, burnout, and self-efficacy) were not presented in the explorative study. Therefore, other factors influencing the relationship between risk perception and PTSD deserve to be profoundly explored.

## Conclusions

At the very core of any public health response, medical staff form the crucible force that must not melt in the heat of the crisis. The lesson we can draw from the COVID-19 for all future outbreaks is that medical staff are neither immune to the virus nor to the psychological stress. Medical staff are vulnerable to PTSD symptoms due to their perceived risks for their surroundings, their raised anxiety, and worsened sleep quality. Hence, the development of PTSD symptoms can be avoided by the candid acknowledgment of the risks and timely implementation of simple protective measures for securing personal safety. Psychological and medical therapies for ameliorating anxiety and sleep quality could also be plausible for deterring the PTSD symptoms, especially the negative cognition and mood. Now that COVID-19 is under control but not eradicated, medical staff's long-term psychological recoveries are in the wake of COVID-19. Further studies are needed to improve the mental protection and preparations of medical staff for the next battle.

## Data Availability Statement

The raw data supporting the conclusions of this article will be made available by the authors, without undue reservation.

## Ethics Statement

The studies involving human participants were reviewed and approved by the ethics committee of the Navy Medical University. The patients/participants provided their written informed consent to participate in this study.

## Author Contributions

QY contributed to the writing of the article and all the analysis. GD contributed to the idea and finally approved the design. WD leaded the whole study, including putting forward this study, getting source and carrying out the study. AC contributed to revise this article and part of statistical analysis. XS contributed to perform the investigation and collection of all data. All authors were accountable for all aspects of the work in ensuring that questions related to the accuracy or integrity of any part of the work are appropriately investigated and resolved, agreed to submit our research result in the article to this journal, and read and approved the final manuscript.

## Conflict of Interest

The authors declare that the research was conducted in the absence of any commercial or financial relationships that could be construed as a potential conflict of interest.
