# Corrigendum: Risk Perception and PTSD Symptoms of Medical Staff Combating Against COVID-19: A PLS Structural Equation Model

**DOI:** 10.3389/fpsyt.2022.890074

**Published:** 2022-04-08

**Authors:** Qianlan Yin, Aibin Chen, Xiangrui Song, Guanghui Deng, Wei Dong

**Affiliations:** Department of Naval Aviation and Operational Psychology, Navy Medical University, Shanghai, China

**Keywords:** risk perceptions, PTSD symptoms, anxiety, sleep quality, PLS model

An author name was incorrectly spelled as “Guanhui Deng.” The correct spelling is “Guanghui Deng.”

The authors apologize for this error and state that this does not change the scientific conclusions of the article in any way. The original article has been updated.

## Publisher's Note

All claims expressed in this article are solely those of the authors and do not necessarily represent those of their affiliated organizations, or those of the publisher, the editors and the reviewers. Any product that may be evaluated in this article, or claim that may be made by its manufacturer, is not guaranteed or endorsed by the publisher.

