# Supplementary material for: Risk Perception and PTSD Symptoms of Medical Staff Combating Against COVID-19: A PLS Structural Equation Model
Source: Front Psychiatry. 2021 Feb 15;12:607612. doi: 10.3389/fpsyt.2021.607612 (PMC7917132; doi:10.3389/fpsyt.2021.607612)
Supplement: Supplementary file 1 [file Data_Sheet_1.PDF]

Supplementary material

1. The model after controlling the effects of age and the model with total sample

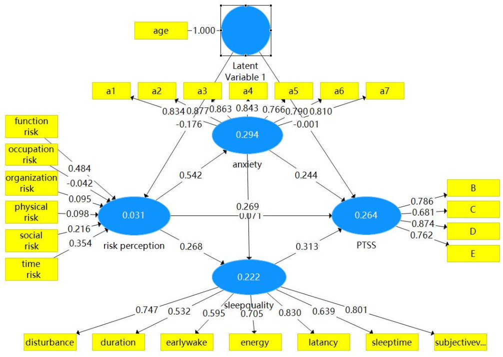

Total Indirect Effects

	Original ...	Sample ...	Standar...	T Statisti...	P Values
Latent Variable 1 -> PTSS	-0.059	-0.061	0.028	2.074	0.038
Latent Variable 1 -> anxiety	-0.095	-0.096	0.040	2.367	0.018
Latent Variable 1 -> risk perception					
Latent Variable 1 -> sleepquality	-0.073	-0.075	0.034	2.163	0.031
anxiety -> PTSS	0.084	0.083	0.027	3.145	0.002
anxiety -> sleepquality					
risk perception -> PTSS	0.262	0.265	0.056	4.716	0.000
risk perception -> anxiety					
risk perception -> sleepquality	0.146	0.146	0.039	3.709	0.000
sleepquality -> PTSS					

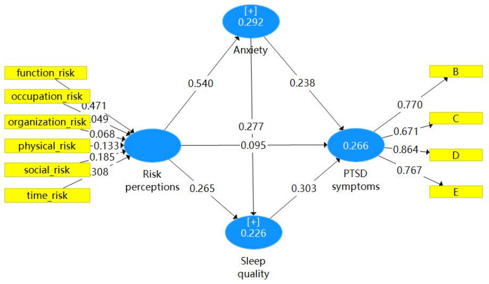

Path Coefficients

	Original ...	Sample ...	Standar...	T Statisti...	P Values
Anxiety_ -> PTSD symptoms	0.238	0.232	0.078	3.031	0.002
Anxiety_ -> Sleep quality	0.277	0.275	0.060	4.634	0.000
Risk perceptions -> Anxiety_	0.540	0.546	0.048	11.180	0.000
Risk perceptions -> PTSD symptoms	0.095	0.105	0.065	1.475	0.140
Risk perceptions -> Sleep quality	0.265	0.278	0.057	4.621	0.000
Sleep quality -> PTSD symptoms	0.303	0.302	0.066	4.615	0.000

Specific Indirect Effects

	Original ...	Sample ...	Standar...	T Statisti...	P Values
Risk perceptions -> Anxiety_ -> PTSD symptoms	0.128	0.128	0.047	2.723	0.007
Risk perceptions -> Anxiety_ -> Sleep quality -> PTSD symptoms	0.045	0.045	0.013	3.367	0.001
Risk perceptions -> Sleep quality -> PTSD symptoms	0.080	0.084	0.027	3.010	0.003
Risk perceptions -> Anxiety_ -> Sleep quality	0.150	0.150	0.036	4.150	0.000

2. Compared with alternative model 1, final model showed a higher level of R^2 for anxiety and sleep quality, and path coefficients for the dependent variable relatively. On the other hand, it was hard to judge final model and alternative model 2 in aspects of R square and the chained indirect effect; therefore, a distribution for the indirect effect on PTSD was evaluated as showed in the figure 1 and figure 2. The bootstrap results presents a relatively bigger indirect effect of anxiety on PTSD through sleep quality (showed in the figure 1). Therefore, given all facets of the model test, the final model was relatively more suited for the prediction.

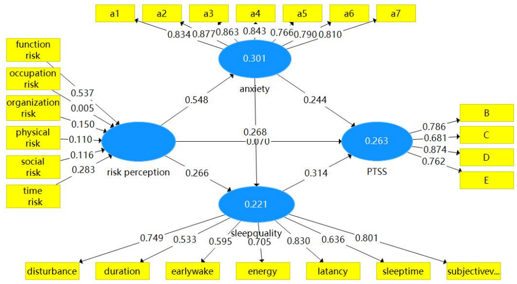

	R Square	R Square Adjusted
PTSS	0.263	0.255
anxiety	0.301	0.298
sleepquality	0.221	0.215

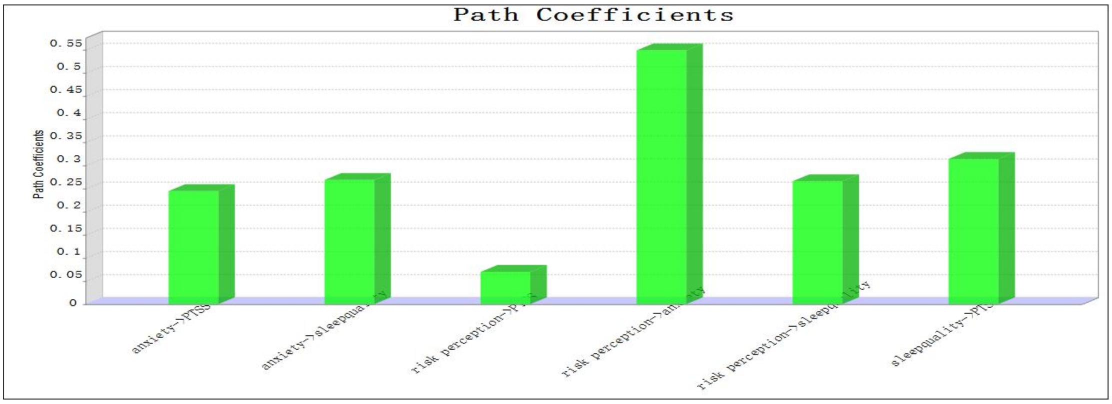

Final model: risk perception→PTSD symptoms

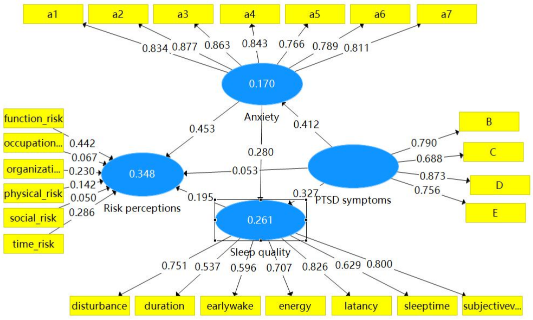

	R Square	R Square Adj...
Anxiety_	0.170	0.167
Risk perc...	0.348	0.341
Sleep qu...	0.261	0.255

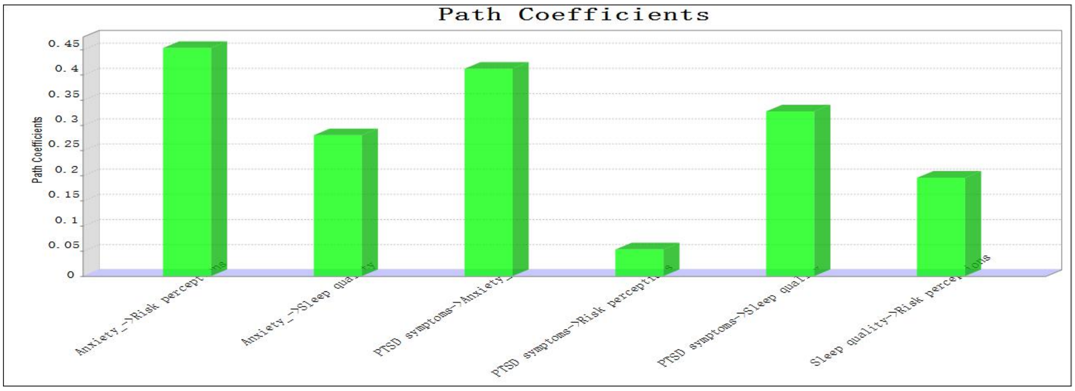

Alternative Model 1: PTSD symptoms→risk perception

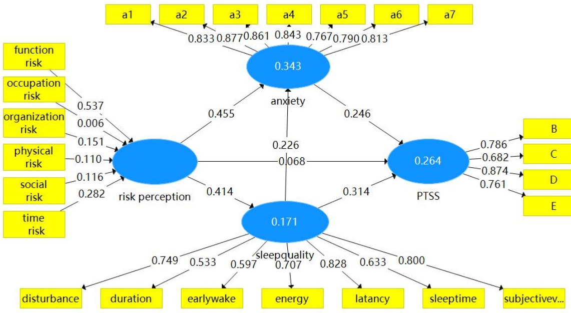

	R Square	R Squar...
PTSS	0.264	0.256
anxiety	0.343	0.338
sleepquality	0.171	0.168

Alternative Model 2: sleep quality→anxiety

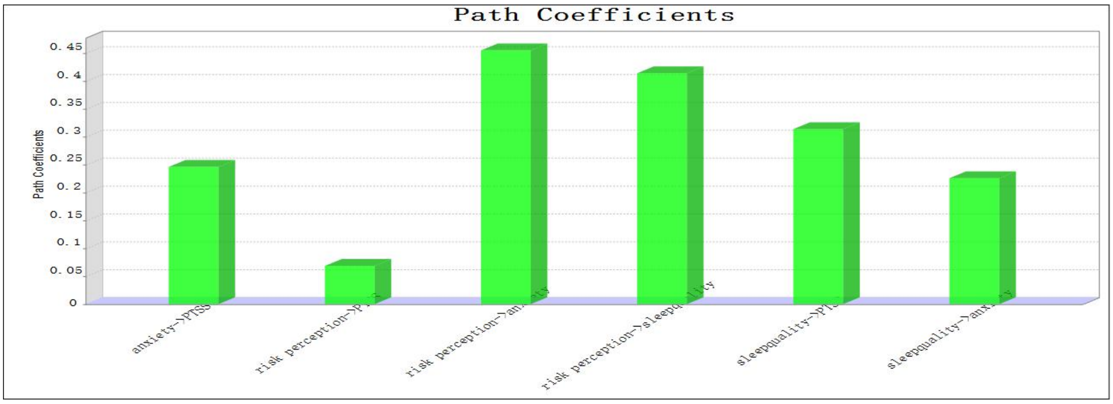

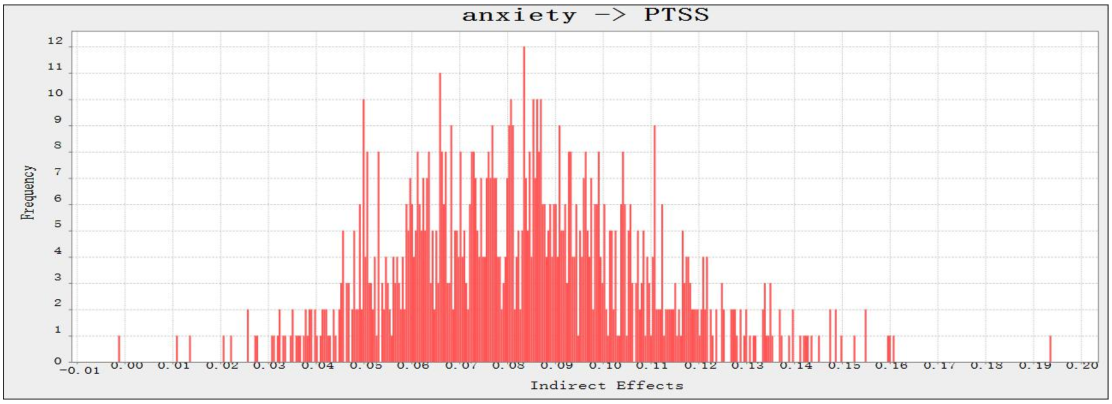

Figure 1

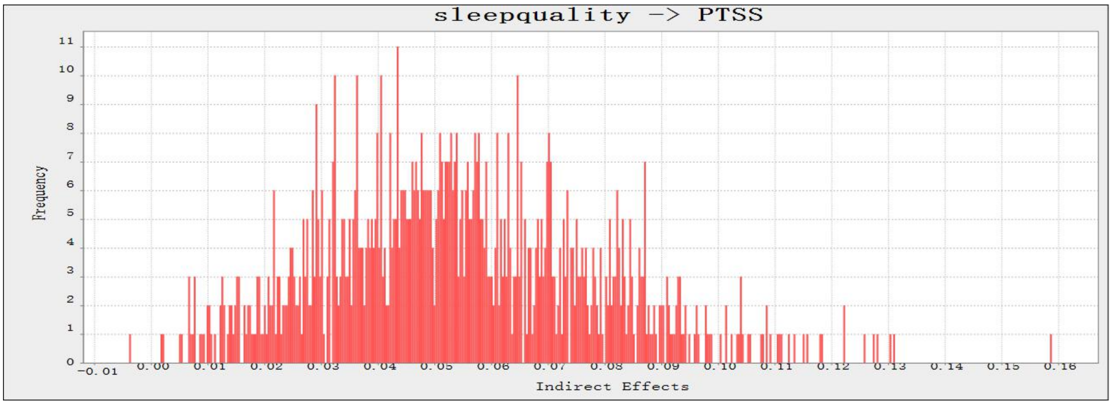

Figure 2
